# Quantitative Fraction Evaluation of Dermal Collagen and Elastic Fibres in the Skin Samples Obtained in Two Orientations from the Trunk Region

**DOI:** 10.1155/2014/251254

**Published:** 2014-09-09

**Authors:** Naveen Kumar, Pramod Kumar, Satheesha Nayak Badagabettu, Keerthana Prasad, Ranjini Kudva, Coimbatore Vasudevarao Raghuveer

**Affiliations:** ^1^Department of Anatomy, Melaka Manipal Medical College, Manipal University, Manipal Campus, Manipal, India; ^2^Consultant Plastic Surgeon, King Abdulaziz Hospital, Al Jouf, Saudi Arabia; ^3^Department of Information Science, Manipal School of Information Science, Manipal University, Manipal, India; ^4^Department of Pathology, Kasturba Medical College, Manipal University, Manipal, India; ^5^Department of Pathology, Yenepoya University, Deralakatte, Mangalore, India

## Abstract

*Background*. Histomorphic evaluation of dermal collagen and elastic fibres was analysed by image analysis technique. The quantification of dermal elements was performed in skin tissues, collected in horizontal and vertical directions from trunk region and discussed under the perspective of consequences of scar related complications. *Materials and Method*. Total number of 200 skin samples collected from 5 areas of trunk region were processed histologically and subjected to tissue-quant image analysis. Statistical analysis involving mean with SEM and paired *t* test by SPSS were employed to the percentage values obtained from image analysis. *Result*. Among the chosen 5 areas of trunk region, abdomen showed the statistically significant difference for both collagen and elastic content between horizontal and vertical orientations (*P< 0.05*), whereas upper back, presternal, and lateral chest areas showed significant difference (*P< 0.05*) only for collagen and groin only for elastic content. *Conclusion*. The differences in the distribution of dermal collagen and elastic fibres in 2 directions of the samples from the same areas might be attributed to final outcome of wound healing process by influencing the appearance and behaviour of scar related complications in the region of trunk.

## 1. Introduction

The mechanical qualities of skin provide a unique arrangement of strength and elasticity. These functional qualities are achieved due to the predominate content of collagen network and to a lesser extent elastin and extracellular matrix substances in the underlying dermis [1]. Functionally, it is believed that these fibres support and nourish the skin to maintain its moisture and elasticity. However, the existence of these proteins in the dermis plays a vital role that determines the appearance and behaviour of scar related problems resulting from the natural process of wound healing. Changes in the morphology of dermis vary among anatomic location, sex, and age of the individual. Children have relatively thin skin, which progressively thickens until the fourth or fifth decade of life when it begins to thin. This thinning is also primarily a dermal change, with loss of elastic fibres, epithelial appendages, and ground substance [2]. Collagen is the most ubiquitous and most durable proteins in nature among the proteins in the body. On other hand, elastin of the skin provides elasticity and this property is made for maximum stretch. Excessive lay down of collagen associated with stretching of scar is more in the skin with abundant elastic content as in children or young adults [3].

The arrangement of dermal collagen, mostly in random form, is designed for its major role in strength and function of the human integument. It appears less parallel in deep dermis when compared to superficial dermis of the normal skin [4] and its bundles show a basket weave-like pattern with random organisation [5].

Dermal elastic network is a strong determinant of skin resilience, texture, and quality but is not sufficiently regenerated following burn injury. In addition to its structural and mechanical roles, elastin has natural cell signalling properties that uphold a varied range of cellular responses including chemotaxis, cell attachment, proliferation, and differentiation. Elastic fibres undergo extensive changes during life. This change may be representing aging or elastotic degeneration due to chronic sun exposure. In very old persons, the fragmentation and disintegration of elastic fibres may be observed [6].

Dermal substitutes are intended to replace damaged dermal tissue in severe burn injuries. This varied nature of dermal connective tissue fibres along with their unequal distributions in the body suggested being probable role in wound healing properties [7]. Quantifying these elements in the skin samples oriented in different directions at the same region also exhibited remarkable asymmetrical distribution. This research study emphasizes similar context in trunk region of the human body. Hence, to find the anatomical cause difference in keloid appearance or scar related complications, the distribution of collagen and elastic tissue between the skin sections taken in 2 orientations perpendicular to each other from trunk region was carried out.

## 2. Materials and Method

### 2.1. Sample Collection

From each area chosen, elliptical (1 × 0.5 cm) skin sections were collected in two directions.

**Figure 1:**
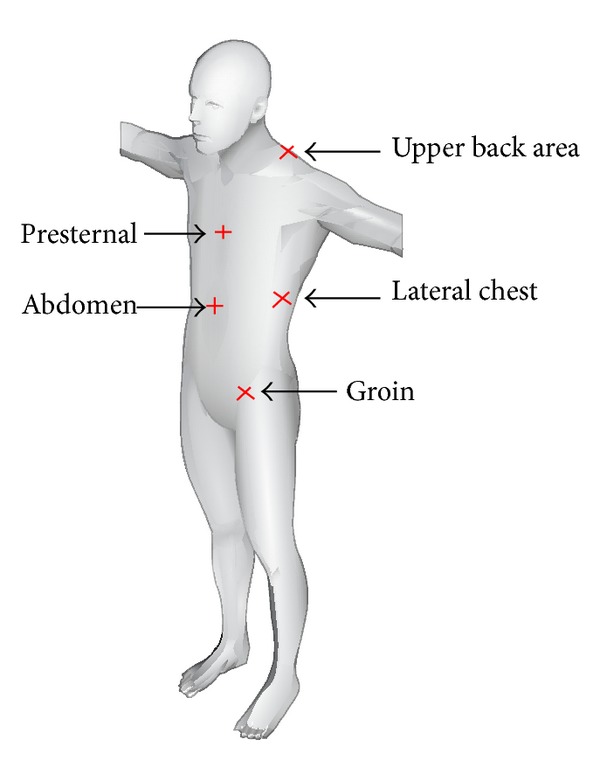
Topographic area on the trunk region where the skin samples were obtained in horizontal and vertical directions (adapted from http://www.3dcadbrowser.com/).

Quantitative fraction of dermal collagen and elastic fibres was evaluated in 200 skin samples of 2 directions obtained from 5 areas of the trunk region from 20 cadavers. Full thickness skin samples were collected from formalin embalmed adult human cadavers of either sex with the age ranging approximately around 55 ± 5 years. From each selected area, elliptical skin sections measuring 1 × 0.5 cm were collected in two directions which were perpendicular to each other. While the first sample was almost “horizontal” to the plane of the region, the second sample was across it and hence termed as “vertical” sample. The topographic site of sample collection was randomly selected and the uniformity was maintained in all the subjects as the following criteria (Figure 1).

*(1) Upper Back Area.* Skin overlying the upper border of trapezius muscle that is between the root of the neck and shoulder joint: sample along the border was “vertical” and across it was “horizontal.”

*(2) Presternal Area.* Samples were collected at the midline over the sternum at the level of the 3rd rib. “Vertical” sample was parallel to plane of sternum and “horizontal” one was across it.

*(3) Lateral Chest Area.* Samples were obtained at the level of the 8th rib in the anterior axillary line. “Horizontal” samples were taken along the direction of the rib; “vertical” samples were perpendicular to it.

*(4) Abdomen.* At the midline of abdomen, 1 cm below the umbilicus: parallel orientation to the linea alba represented as “vertical” and section across it as “horizontal.”

*(5) Groin.* Over the midpoint of inguinal ligament: Sample taken along the direction of inguinal ligament served as “horizontal” direction, while perpendicular to it as “vertical”.

### 2.2. Histological Processing

Skin samples were processed under histological techniques to obtain paraffin sections. These sections were treated through series of solutions till hydration. A special stain Verhoeff-van Gieson (VVG) was employed to selective demonstration of collagen and elastic fibres [8]. These slides were observed under light microscope to ascertain normal histological pattern of dermis. From each section stained, 3 photomicrographs were collected under 20x magnification using inverted phase contrast microscope attached with ProgRes CapturePro 2.1, Jenoptik, microscopic camera. Each image was captured with the resolution of 694 × 516 VGA for the image analysis by “tissue-quant” method [7]. Images were collected just beneath the epidermis including both papillary and reticular layers of the dermis in a random fashion.

### 2.3. Image Analysis

**Figure 2:**
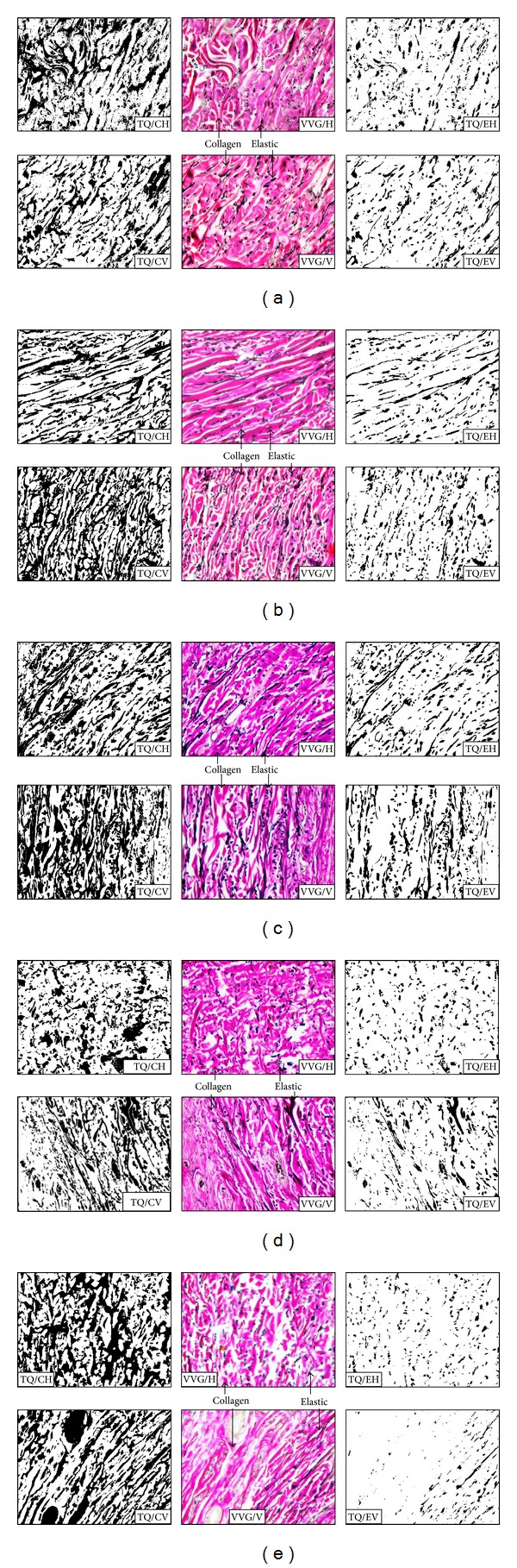
Photograph showing the pattern of collagen (stained pink) and elastic (black stain) fibres as in Verhoeff-van Gienson (VVG) stain (×20) in horizontal (VVG/H) and vertical (VVG/V) samples of upper back (a), presternal (b), lateral chest (c), abdomen (d), and groin (e) areas of trunk region. Segmentation of collagen and elastic fibres by tissue-quant (TQ) image analysis is shown in adjacent photographs. TQ/CH and TQ/EH: for collagen and elastic fibres in horizontal section, respectively, and TQ/CV and TQ/EV: as in vertical sections, respectively.

Image analysis of the VVG sections based on their property of colours acquired by the stain employed was done by the software “tissue-quant” version 1.0. The tissue-quant software work on the principle of scoring the number of pixels assigned to the shades of the colour to be analysed. The software has the option of obtaining strength of staining in terms of a colour score. Selection of positively stained area in the image is measured by means of number of pixels assigned by it. This corresponds to the quantitative fraction of the structure to be measured. This process is prerequisite by the segmentation of colour and its shades of interest from the rest of the colours (Figure 2). Total number of pixels corresponding to the colour of interest is then converted to percentage value by proper calculation [9]. The results obtained from all 3 images of each section were aggregated for further statistical evaluation.

### 2.4. Statistical Analysis

Mean value with standard error of mean was calculated from the percentage values of quantitative fraction; in addition, paired sample *t* test with 95% confidence level was employed to compare all the variables between two different directions separately for collagen and elastic fibres. The *P* < 0.05 is considered to be statistically significant.

## 3. Results

Results of quantitative fraction of dermal collagen and elastic fibres measured based on percentage area occupied by them in the samples obtained in horizontal and vertical direction with their level of significance are tabulated in Table 1.

Upon analysis of content of dermal elements in the trunk region by image analysis method, it has been observed that the consistency of collagen remained more dominant in horizontally oriented sections than its vertical counterpart in all the areas tested. The difference in collagen distribution between 2 directions was also found to be statistically significant (*P* < 0.05) in all areas tested except in the groin area. Contrary to this, the elastic fibre content exhibited an asymmetric pattern of predominance as far as orientations of the section were concerned. It was observed to be great in the vertically oriented than in horizontal sections of upper back, lateral chest, and groin area with the significant difference in groin region (*P* < 0.05) only. On the other hand, in presternal and abdomen areas, the elastic dominance was found to be more in horizontally directed samples with the significant difference at the abdomen area (*P* < 0.05).

## 4. Discussion

Tissue injury resulting in irreversible cell death and connective tissue disruption initiates the repair process. Wounds heal by scarring where a new cell population resides in a newly deposited connective tissue matrix. In certain regions of the body, surgical wounds heal with a better and less noticeable scar if they are lying in a particular direction. This is because of number of factors including skin tension and naturally formed wrinkle lines. Skin tension is due to protrusion of underlying structures and direction of underlying muscle and joint movements. Though the anatomists and surgeons have attempted to produce a body map to indicate the best direction for elective incision to obtain the most aesthetic scar, these maps frequently differ in regions, specifically on face [10].

Histomorphic assessment of collagen and elastic fibres of abdominal skin after abdominoplasty procedure performed on obese females for weight loss revealed the deficiency of collagen fibre network mainly in epigastrium region without damaging elastic fibres [11]. This is possibly because of opposed action of elastic fibres by stretching the skin, which in turn exerts the stretching force on the scar [7].

Hypertrophic scarring is mostly developed in wounds at anatomic locations with high tension, such as shoulders, neck, presternum, knees, and ankles [12, 13], whereas risk of keloid formation is higher at anterior chest, shoulders, earlobes, upper arms, and cheeks [14]. Scar stretching occurs most frequently in the lower third of the scar overlying the xiphisternum and extending onto the abdomen [15].

Although the importance of dermal connective tissue fibres is widely approved, no uniform evaluation method was available for the reliable quantification in terms of their amount of area of occupancy. For research that focuses on wound healing and scar formation, polarised light is the established method for the evaluation of these structures. However, an image analysis technique reported to be more accurate than observer ratings [15]. Thus, we employed a simple and reliable-method of quantification based on their area of occupancy in the field of image measured by tissue-quant software.

Concepts of skin lines in aesthetic approach gained much importance, though there was no clear explanation for individuals view. However, universal agreement as widely accepted is that if a scar follows a certain direction, a better scar will be produced than if it is at right angles to that direction. Lack of evidence for this fact made each researcher attempt the explanation based on 3 factors of skin tensions: physical, anatomic, and functional or experimental (empirical) methods [16].

This actuality is being supported by plastic surgeons, as in the living individuals gaping of the wound may be produced by anatomical (due to dermal elastic content) or functional (at joints) or physical (closure under tension) reasons. The later factor is dependent on surgeons, as, whenever skin is closed under tension, the scar is often unacceptable due to physical reason. Similarly, over the joints, a horizontal scar heals better due to less physical tension on the healing wounds, irrespective of anatomical differences in elastic fibers in different orientation.

On the other hand, the concept of Langer's line has been established by the observation of gaping (elliptical shaped opening) on the skin by the puncture wound made at particular direction. The direction which produced less gaping was considereda Langer's line of that particular area, without any explanation for this [16]. And also, one can explain the difference in collagen content in 2 different directions. The collagen content is meant for the strength of the structure or scar. Therefore, collagen content will be often more on one or both directions (horizontal/vertical) depending on the repeated stress due to associated elastic fiber content, physical stretching, or functional reason. Accordingly, present findings can be justified as follows.

*In the Upper Back Area.* Anatomical cause is subsided, as there was no significant difference in the elastic content between 2 directions. Hence, it is expected that anatomical factors will not be important in determination of quality of scars at the upper back area. It is in accordance with general experience of surgeons that, at this area, scar in any direction is usually unaesthetic. But, due to functional reason, repeated stretching over the back, in particular, direction as a result of flexion of spines in daily activities, might produce more stress in horizontal direction. Therefore, collagen content along the horizontal direction is supplementary to augment the strength of dermis as evident from our findings.

*Presternal Area.* Here, too, anatomical factor is not playing significant role in the formation of unacceptable scar, as there was no significant difference in the elastic content between 2 directions. Thus, Langer's line concept for aesthetic scar will not be acceptable in presternal area as is the general experience of surgeons. However, due to functional reason, repeated stretching in upward and oblique direction by the shoulder movements, the collagen content is expected to be more in horizontal direction (H > V) than in vertical one.

*Lateral Chest Area.* Anatomical factor at lateral chest area is also not playing major role in the formation of unacceptable scar, due to elastic content. The functional cause, during each respiration leading to chest expansion, produces maximum stretch in vertical direction and therefore the collagen content is significantly higher in vertical direction than in its horizontal counterpart.

*Abdomen.* At abdomen region, both the collagen and elastic content are significantly higher in horizontal direction, indicating that more collagen in horizontal direction cannot be explained due to anatomical reason. Continuous abdominal movement due to pushing down of abdominal content by the diaphragmatic movement may be appropriate functional reason. Increased collagen in horizontal plane explains more stress along the horizontal direction which is not due to anatomical reason as explained by elastic fiber content. Hence, increased elastic content in horizontal direction in abdomen area is responsible for an unacceptable scar in vertical direction. Thus, Langer's line concept holds well over the abdomen.

*Groin.* Elastic fiber content is extremely higher on vertical direction with the statistical significant difference between 2 directions. Thus, scar along the crease is expected to be under stress, giving rise to unaesthetic result, which is contrary to routine observation by the surgeons. This may be explained by nullified action of elastic fibre by flexed posture of hip joint during most of the activities (functional). The collagen content difference is insignificant.

*Conclusion.* Often, collagen content will be more in a particular direction depending on the repeated stress due to associated elastic fibre content, physical stretching or functional reason. These facts are confirmed with the results of present research study by the asymmetric distribution of dermal collagen and elastic fibre content along horizontal and vertical orientations of the skin samples obtained.

## Figures and Tables

**Table 1:** Mean quantitative fraction (%) with SEM and the result of paired sample *t* test between 2 directions for collagen and elastic fibre quantity measured by tissue-quant image analysis.

Topographic area	Collagen			Elastic		
	Horizontal section	Vertical section	*P *value	Horizontal section	Vertical section	*P* value
	Mean (%) with SEM	Mean (%) with SEM		Mean (%) with SEM	Mean (%) with SEM	
Upper back	51.9 ± 1.6	49.2 ± 1.8	**0.03** ∗	9.7 ± 0.6	10.5 ± 1	**0.25**
Presternal	52.3 ± 2.1	47.5 ± 1.8	**0.01** ∗	13.6 ± 1	12.4 ± 0.9	**0.18**
Lateral chest	54.1 ± 1.8	49.7 ± 2.2	**0.02** ∗	10.3 ± 0.8	11.6 ± 0.8	**0.16**
Abdomen	52.1 ± 1.9	47.1 ± 1.9	**0.009** ∗	14.7 ± 1.2	12.8 ± 1.1	**0.04** ∗
Groin	51.5 ± 2.2	49.8 ± 2.2	**0.25**	11.5 ± 0.8	13.4 ± 1	**0.02** ∗

*Indicates statically significant (*P* < 0.05) difference between “horizontal” and “vertical” directions of samples taken.

## References

[1] Peacock EE (1984). *Structure Synthesis and Interaction of Fibrous Protein and Matrix in Wound Repair*.

[2] Burns DA, Breathnach SM, Cox N, Griffiths CE (2004). *Rook's Textbook of Dermatology*.

[3] Berman B, Viera MH, Amini S, Huo R, Jones IS (2008). Prevention and management of hypertrophic scars and keloids after burns in children. *Journal of Craniofacial Surgery*.

[4] van Zuijlen PPM, Ruurda JJB, van Veen HA (2003). Collagen morphology in human skin and scar tissue: no adaptations in response to mechanical loading at joints. *Burns*.

[5] Linares HA, Herdon DN (1996). Pathophysiology of the burn scar. *Total Burn Care*.

[6] Elder DE (2009). *Lever’s Histopathology of the Skin*.

[7] Naveen K, Pramod K, Keerthana P, Satheesha NB (2012). A histological study on the distribution of dermal collagen and elastic fibres in different regions of the body. *International Journal of Medicine and Medical Sciences*.

[8] John DB (2002). Theory and practice of histological techniques (5th edition). *Marilyn Gamble*.

[9] Prasad K, Kumar PB, Chakravarthy M, Prabhu G (2012). Applications of “TissueQuant”—a color intensity quantification tool for medical research. *Computer Methods and Programs in Biomedicine*.

[10] Standring S (2005). *Gray’s Anatomy: The Anatomical Basis of Clinical Practice*.

[11] From L, Assad D, Jeffers JD, Englis MR (1993). Neoplasms, pseudo-neoplasms and hyperplasia of supporting tissue origin. *Dermatology in General Medicine*.

[12] Hawkins HK, Herndon DN (2007). Pathophysiology of the burn scar. *Total Burn Care*.

[13] Niessen FB, Spauwen PHM, Schalkwijk J, Kon M (1999). On the nature of hypertrophic scars and keloids: a review. *Plastic and Reconstructive Surgery*.

[14] Elliot D, Cory-Pearce R, Rees GM (1985). The behaviour of presternal scars in a fair-skinned population. *Annals of the Royal College of Surgeons of England*.

[15] van Zuijlen PPM, de Vries HJC, Lamme EN (2002). Morphometry of dermal collagen orientation by Fourier analysis is superior to multi-observer assessment. *The Journal of Pathology*.

[16] Borges AF (1984). Relaxed skin tension lines (RSTL) versus other skin lines. *Plastic and Reconstructive Surgery*.

