# Erratum to “Quantitative Fraction Evaluation of Dermal Collagen and Elastic Fibres in the Skin Samples Obtained in Two Orientations from the Trunk Region”

**DOI:** 10.1155/2015/596985

**Published:** 2015-01-05

**Authors:** Naveen Kumar, Pramod Kumar, Satheesha Nayak Badagabettu, Keerthana Prasad, Ranjini Kudva, Coimbatore Vasudevarao Raghuveer

**Affiliations:** ^1^Department of Anatomy, Melaka Manipal Medical College, Manipal University, Manipal Campus, Manipal, India; ^2^Consultant Plastic Surgeon, King Abdulaziz Hospital, Al Jouf, Saudi Arabia; ^3^Department of Information Science, Manipal School of Information Science, Manipal University, Manipal, India; ^4^Department of Pathology, Kasturba Medical College, Manipal University, Manipal, India; ^5^Department of Pathology, Yenepoya University, Deralakatte, Mangalore, India

In the paper titled “Quantitative Fraction Evaluation of Dermal Collagen and Elastic Fibres in the Skin Samples Obtained in Two Orientations from the Trunk Region” in page 5 under Discussion Section paragraph 3 last line reference 15 should be read as reference 14 as follows:

“Scar stretching occurs most frequently in the lower third of the scar overlying the xiphisternum and extending onto the abdomen [14].”

In page 6 paragraph 3 one sentence (quoted below) gives wrong impression and should be corrected as follows:

“The functional cause (the expansion of chest during respiration) leads to increase in the girth and hence stretch will be in horizontal direction. This necessitates more significant increment of collagen fiber content in horizontal direction than in its vertical direction (H > V) (Table 1).”

